# An integrated structural intervention to reduce vulnerability to HIV and sexually transmitted infections among female sex workers in Karnataka state, south India

**DOI:** 10.1186/1471-2458-11-755

**Published:** 2011-10-02

**Authors:** Vandana Gurnani, Tara S Beattie, Parinita Bhattacharjee, CFAR Team, HL Mohan, Srinath Maddur, Reynold Washington, Shajy Isac, BM Ramesh, Stephen Moses, James F Blanchard

**Affiliations:** 1 Karnataka Health Promotion Trust, IT/BT Park, 4th and 5th Floor, #1-4/, Rajajinagar Industrial Area, Behind KSSIDC Administrative Office, Rajajinagar, Bangalore 560 044, India; 2 Department of Global Health and Development, London School of Hygiene and Tropical Medicine, Keppel Street, London, WC1E 7HT, UK; 3 Centre for Advocacy and Research, New Delhi, India; 4 Department of Community Health Sciences, University of Manitoba, 730 William Avenue, Winnipeg, R3E 0W3, Canada; 5 Department of Medical Microbiology, University of Manitoba, 730 William Avenue, Winnipeg, R3E 0W3, Canada

## Abstract

**Background:**

Structural factors are known to affect individual risk and vulnerability to HIV. In the context of an HIV prevention programme for over 60,000 female sex workers (FSWs) in south India, we developed structural interventions involving policy makers, secondary stakeholders (police, government officials, lawyers, media) and primary stakeholders (FSWs themselves). The purpose of the interventions was to address context-specific factors (social inequity, violence and harassment, and stigma and discrimination) contributing to HIV vulnerability. We advocated with government authorities for HIV/AIDS as an economic, social and developmental issue, and solicited political leadership to embed HIV/AIDS issues throughout governmental programmes. We mobilised FSWs and appraised them of their legal rights, and worked with FSWs and people with HIV/AIDS to implement sensitization and awareness training for more than 175 government officials, 13,500 police and 950 journalists.

**Methods:**

Standardised, routine programme monitoring indicators on service provision, service uptake, and community activities were collected monthly from 18 districts in Karnataka between 2007 and 2009. Daily tracking of news articles concerning HIV/AIDS and FSWs was undertaken manually in selected districts between 2005 and 2008.

**Results:**

The HIV prevention programme is now operating at scale, with over 60,000 FSWs regularly contacted by peer educators, and over 17,000 FSWs accessing project services for sexually transmitted infections monthly. FSW membership in community-based organisations has increased from 8,000 to 37,000, and over 46,000 FSWs have now been referred for government-sponsored social entitlements. FSWs were supported to redress > 90% of the 4,600 reported incidents of violence and harassment reported between 2007-2009, and monitoring of news stories has shown a 50% increase in the number of positive media reports on HIV/AIDS and FSWs.

**Conclusions:**

Stigma, discrimination, violence, harassment and social equity issues are critical concerns of FSWs. This report demonstrates that it is possible to address these broader structural factors as part of large-scale HIV prevention programming. Although assessing the impact of the various components of a structural intervention on reducing HIV vulnerability is difficult, addressing the broader structural factors contributing to FSW vulnerability is critical to enable these vulnerable women to become sufficiently empowered to adopt the safer sexual behaviours which are required to respond effectively to the HIV epidemic.

## Background

Structural factors, including social, cultural, economic, political, legal and environmental factors, are known to affect individual risk and vulnerability to HIV, and to operate at several different societal levels (individual, interpersonal, community, culture and policy) [1]. HIV prevention in the context of female sex work continues to be a high priority globally, particularly in settings where heterosexual contact is the main transmission mode, but the epidemic has yet to become generalised [2-4]. Early interventions with female sex workers (FSWs) typically relied on individual behaviour change involving peer education, condom promotion and provision of sexual health services [5], and many were limited in scale [6]. Exceptions to this included the 100% condom programme in Thailand which was a government-led structural intervention, targeting the mainly brothel-based sex worker population and their clients [7]; and the Sonagachi programme in Kolkata, India, which provided one of the first examples of a rights-based FSW HIV prevention programme, focusing on the community mobilisation and empowerment of sex workers alongside engagement with power structures [8-10]. Although these examples differed in approach, both recognised the need to address broader structural factors to create an enabling environment within which individual behaviour change could occur. More recently, a comparison of two structural intervention models with FSWs in the Dominican Republic found that an intervention which combined community mobilisation with government policy was more effective, and more cost-effective, than community mobilisation alone in reducing HIV and STI risk among FSWs [11,12].

Karnataka state in South India has a population of approximately 60 million and, with an adult HIV prevalence of approximately 1% in several districts, ranks in the top four states in India with regard to epidemic severity [13,14]. Heterosexual contact is the main route of transmission, with commercial sex work a key area of concern. The FSW population has been estimated to number over 100,000, and HIV rates have reached more than 25% among FSWs in some districts [15,16]. In contrast to Thailand, Kolkata and the Dominican Republic, where brothel-based sex work largely features, the majority of FSWs in Karnataka are street- and home-based, working in rural as well as urban settings [17,18].

Prior to 2003, condom availability throughout Karnataka was low [19], and HIV prevention programmes targeting female sex workers were operating on a small scale in only a few of the 27 districts. As part of *Avahan*, the India AIDS Initiative, funded by the Bill & Melinda Gates Foundation, which works with core and bridging populations in 83 districts in six Indian states [20], the Karnataka Health Promotion Trust (KHPT) was established in 2003 with the aim of rapidly scaling up HIV prevention efforts through targeted interventions in 18 of Karnataka's then 27 districts. Programme implementation at the community level was through non-governmental organizations (NGOs) or community-based organizations (CBOs), and included a rights-based approach [21]. The gradual handover of ownership to the FSW community and the Indian government has now begun, with 30% of the districts transitioned in April 2011, and plans for the remainder to be transitioned by April 2012. This process has been managed by KHPT, which will continue to provide post-transition support for another one to two years to help ensure the maintenance of programme quality.

In the early stages of the programme, needs assessment workshops were conducted with FSWs in each district, where women were asked about the problems and challenges they were facing in their lives. Key themes to emerge from these workshops were that issues relating to poverty (housing, food and schooling insecurities), stigma and discrimination, and violence and harassment, were major and common concerns of FSWs and were contributing to their vulnerability to HIV. For FSWs to prioritise their health needs, it became clear that, alongside the provision of standard programme initiatives including peer education, condom promotion and sex-worker specific sexual health services, these context-specific broader structural barriers contributing to FSW vulnerability would also need to be addressed. An enabling environment needed to be created in which HIV prevention programmes and services could be embedded [22-24].

**Figure 1:**
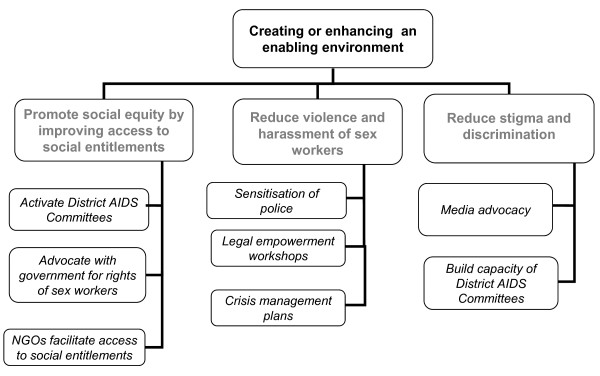
**Framework for addressing structural barriers contributing to female sex worker vulnerability to HIV/STIs**.

We have previously reported on the impact of the HIV prevention programme in terms of reductions in violence, increases in condom use, and reductions in HIV and STI prevalence among the female sex worker population [16,21,25,26]. In this paper we provide details of the community components of the programme, how we have worked to bring the FSW community in Karnataka together through processes of collectivisation, community mobilisation and empowerment and how, in partnership with the FSW community, we have engaged with policy makers (government), secondary stakeholders (the police, the media and human rights lawyers), and primary stakeholders (FSWs themselves) to address the broader structural barriers which contribute to FSW vulnerability to HIV and other sexually transmitted infections (STIs) (Figure 1).

## Programme design and implementation

### Collectivisation, community mobilisation and empowerment of FSWs

**Figure 2:**
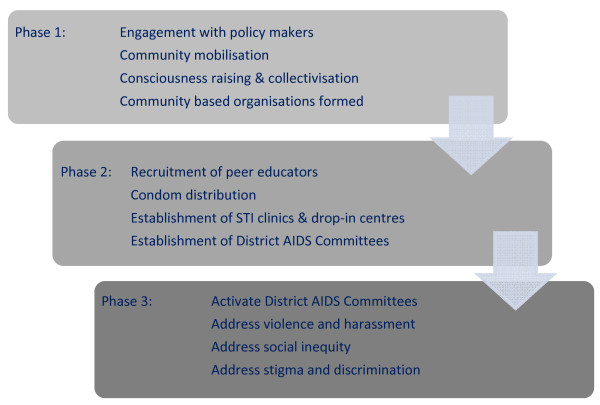
**Phasing of activities in addressing structural barriers contributing to female sex worker vulnerability to HIV/STIs**.

The collectivisation process involves developing critical thinking among FSWs, mobilising and collectivizing the community, and engaging them in processes leading to organisational development [27]. Organising FSWs into support groups and community-based organisations can help the community collectively challenge structural barriers contributing to their vulnerability to HIV/STI, including stigma, discrimination, violence, harassment and social inequity [9,28]. Following baseline mapping, the programme began the process of community mobilisation, bringing FSWs who were scattered across cities and rural areas together into a community (Figure 2). Peer educators were recruited from sex work sites and, following consultation with FSW communities, sexual health clinics and drop-in centres were established in all project areas. Drop-in centres provide a safe space where FSWs can meet and access practical and emotional support from each other and the programme, and facilities and activities include literacy classes, shower facilities, hot food, and weekly meetings where the community can raise and collectively address difficulties in their lives [21].

To facilitate the empowerment process, KHPT actively encouraged and supported the formation of FSW-owned community-based organisations throughout Karnataka, and worked to involve the FSW community in all aspects of HIV prevention programme design and implementation, including building the capacity of and gradually handing over management of the programmes to elected FSW representatives. In addition, FSWs, including those living with HIV, were recruited from across the state and provided with training to enable them to actively participate in District AIDS Committee (DAC) meetings (described below), and to co-facilitate sensitisation and HIV awareness training workshops to heads of government departments, the police and journalists (described below).

### Engagement with policy makers

To support the effective implementation of the HIV prevention programme and to help address FSW issues of social inequity, stigma, discrimination, violence and harassment, KHPT advocated successfully with the government of Karnataka for the formation of District AIDS Committees (DACs) in each district. These committees were chaired by the Deputy Commissioner (the senior government official in the district), and comprised the heads of all government development departments and the chief of the police. Importantly, to ensure representation of the community, orders were issued that these committees must also include an HIV-positive person and a female sex worker. The role of the committees is to integrate HIV prevention and care activities into all governmental programmes, and to facilitate implementation of government and donor funded HIV/AIDS programmes.

KHPT provided logistic support for District AIDS Committee meetings, and ensured coordination between the NGO partners and local health officials organising meetings. Senior representatives of KHPT attended the District AIDS Committee meetings and made presentations to the committees regarding the HIV situation in the state and the district, details of the programmes being implemented in the district, and the support needed from the different government departments. KHPT also prepared draft minutes of meetings, and followed up on the issuance of meeting proceedings and compliance with decisions taken in the meetings.

In order for the heads of government and police departments to understand the local HIV/AIDS epidemic and the role sex work can play in transmission, and to develop empathy and understanding about the lives of FSWs and the detrimental impact structural barriers can have on HIV prevention programming, it became apparent that they would require training around these issues. KHPT again advocated with the government, who in turn ordered the heads of the key government development and police departments to attend two-day sensitisation training workshops, designed and delivered by KHPT in partnership with the government and the FSW community.

### Addressing stigma and discrimination

India is a conservative society, where sex outside of marriage is widely disapproved of, and the context in which many women enter the sex trade is rarely understood by the public [29]. Karnataka has thirteen mainstream state-level newspapers (six in *Kannada*, the local state language, and seven in English), and five to ten local newspapers in each district. The media can play a key role in generating awareness on HIV/AIDS related issues, but monitoring of newspaper reports in 2005/2006 found that few provided accurate information, and articles on FSWs largely focused on negative events, such as raids and arrests. To help address stigma and discrimination against FSWs, in partnership with KHPT and the Centre for Advocacy and Research (CFAR), FSWs engaged with senior journalists and sought their support in collaboratively developing a sensitization curriculum on HIV and sex work for journalists.

Spokesperson training was provided to FSWs, including those living with HIV/AIDS who were willing to disclose their HIV status and discuss their experiences with the media, and one-day workshops were delivered to over 950 journalists across the state. In a break with tradition, each workshop was inaugurated with the chief guest shaking hands with an HIV positive person, providing a good photographic opportunity for journalists and sending a powerful message to readers. In 2007, KHPT facilitated a visit by the Chief Minister of Karnataka to a small village in northern Karnataka, where he spent a night with a family living with HIV/AIDS. This event received enormous local, national and international press coverage, with over 70 media reports in Karnataka alone.

### Addressing violence and harassment

(i) Sensitisation of the Police

Sex work *per *se is not illegal in India, but many police and FSWs wrongly understand this to be the case, with the police frequently demanding sex acts in exchange for non-arrest, as well as arresting and beating sex workers and extorting bail monies [30]. Violence, harassment and the fear of arrest can undermine HIV prevention programming in several ways [31-33]. Coerced sex is rarely protected, and the fear of violence can deter sex workers from negotiating condom use and from accessing sexual health services [34-37]. In addition, experiencing violence and harassment can negatively impact on the mental health and self-esteem of women [38], which in turn can reduce their will and/or ability to negotiate condom use or to access STI services. Where sex work occurs in public places, the fear of arrest can lead to hurried encounters, with little time for condom negotiation [39]. In addition, condoms carried by FSWs can be used as evidence of sex work by police.

Following continuous advocacy by KHPT with the Police and Home Affairs Departments, The Director-General and Inspector-General of Police of Karnataka issued instructions to all police personnel in the State stating that: (i) while booking cases under the Immoral Traffic Prevention Act, 1956, efforts should be made to book the traffickers, pimps or other agents; (ii) women in sex work should be viewed with sympathy and the anomalous enforcement of the Act should be corrected; and (iii) all allegations of police harassment or connivance should be promptly enquired into and strict action taken against the offending officers. In addition, to enable police officers to correctly understand the laws pertaining to sex work, to generate understanding about the lives of FSWs, and to address FSW violence and harassment by the police, the Director-General and Inspector-General of Police ordered all police officers to attend one-day training workshops held at police stations across Karnataka. The content of these workshops was developed following inputs from experts from the field, including FSWs, and the workshops were delivered by core training teams comprising the Deputy Superintendent of Police, a peer educator (FSW) with training skills, a human rights lawyer and KHPT staff.

(ii) Legal Empowerment Workshops

To help appraise FSWs as to their rights regarding sex work and arrest, state-wide legal empowerment workshops were rolled out in partnership with human rights lawyers to the FSW community in every district. These workshops included working through real-life sex worker case studies to help FSWs understand how the law applies to various situations. In addition, in partnership with the police, tours were conducted around local police stations to allow FSWs to meet their local police officers and understand the process for police bookings, and for filing complaints against the police.

(iii) Crisis Management Teams

In order to respond to FSWs during emergencies, such as incidents of physical or sexual violence and wrongful arrest, twenty-four hour crisis response teams were established in each area comprising NGO staff, FSWs and human rights lawyers. Emergency mobile phone numbers were widely distributed among the FSW community, providing sex workers with an immediate response in times of crisis, as well as advocating on the victim's behalf to pursue justice against perpetrators of violence [25].

### Addressing social inequity

Social inequities adversely impact upon the lives of female sex workers. They often are illiterate and do not have a husband to support them financially, and frequently face a daily struggle to earn enough money to survive [29,37]. Clients often offer more money for unprotected sex, and, in the context of extreme poverty, negotiating condom use as well as taking time to access sexual health services can be less important than the immediate need for sex workers to provide for themselves and their families. Although government benefits, including free housing, food and schooling schemes, are available to help the poorest in Indian society, structural barriers often prohibit FSW access to these schemes. The application procedure requires levels of literacy beyond that of many FSWs, and discrimination of FSWs by government officials can lead to the explicit denial of those FSWs who do apply. The requirement of a residential address to access food ration cards (providing 10 kg of rice per month), and the requirement of a child's father to be present to access free schooling, present additional structural barriers.

To help overcome these barriers, following advocacy by KHPT, the Karnataka government was persuaded to issue an order prohibiting the discrimination of FSWs by government officials. In addition, a special housing scheme has been created for FSWs, PLWHAs and *Devadasi *(a group of sex workers in northern Karnataka who are part of a long-standing tradition in which adolescent girls are dedicated to gods and goddesses and subsequently inducted into sex work) [40]. To help empower the sex worker community to access state benefits previously denied to them by government officials, KHPT conducted social entitlement workshops with FSWs in all districts, explaining to them the social entitlements they were eligible for. Women were also supported through the application process.

## Methods

To assess the impact of these structural interventions on FSW collectivisation, mobilisation and empowerment, as well as on FSW experience of stigma, discrimination, violence, harassment and social equity over time, we examined data from routine programme management information systems and from daily monitoring of newspapers in Karnataka.

### Management information systems

Standardised, routine programme monitoring indicators, part of programme management information systems, have been collected monthly from each of the 18 districts in Karnataka where KHPT operates since December 2005, with the quality of this data assured from January 2007. Data are collected on service provision, service uptake and community activities. This information system is used to identify where changes need to be made to improve the operation of the HIV prevention programme at multiple levels [41,42]. The key indicators collected cover infrastructure (e.g. number of clinics and drop-in centres), human resources (e.g. number of peer educators), and service utilization (e.g. number of condoms distributed and number of individuals visiting a clinic in a month). Data are also captured on peer educator interactions, utilization of STI services, and operational and infrastructure aspects reported by the NGOs. Integrity of data is ensured by the implementing partners.

### Media monitoring

Since 2005, daily tracking of news articles concerning HIV/AIDS and FSWs has been conducted manually by media monitors in selected districts, including the capital, Bangalore. In 2006 and 2008, news reports were monitored from nine state-wide publications (five in *Kannada*, the local language, and four in English), and district-level newspapers from the cities of Bangalore, Belgaum, Hubli, Dharwad, Gulbarga and Shimoga districts.

Data collected on "tracked" news reports included the name of newspapers, date, page number, photographs, article themes, stories or quotes from trained spokespersons (such as FSWs), reporters writing on specialized subjects such as stigma, and newspapers concentrating on negative aspects of HIV/AIDS/sex work to create sensational news.

### Ethical approval

Ethical approval for this study was granted by the Institutional Review Board of St. John's Medical College in Bangalore, India.

## Results

### Collectivisation, community mobilisation and empowerment of FSWs

**Figure 3:**
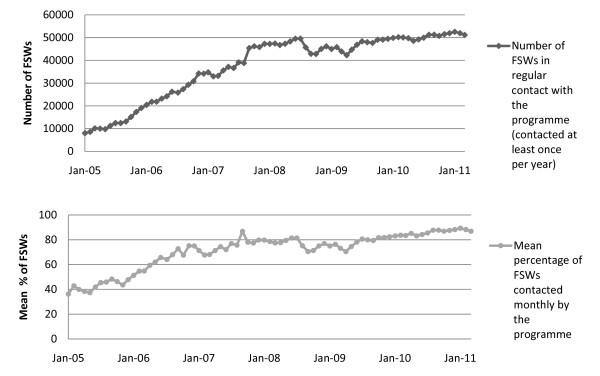
**Estimated number of female sex workers with regular monthly programme contact and mean percentage of female sex workers contacted monthly by the programme, in 18 districts in Karnataka state, January 2005 to March 2011**.

**Figure 4:**
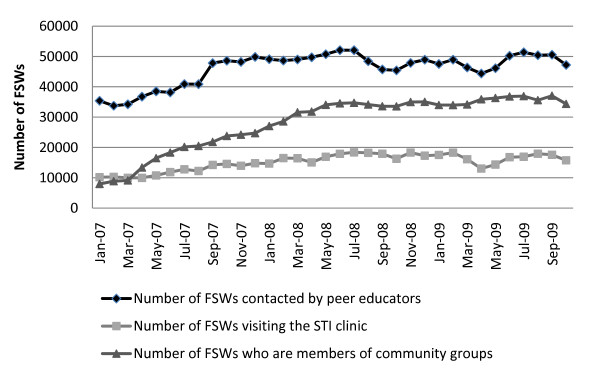
**Increases over time in indices of community mobilisation of female sex workers, January 2007-October 2009**.

The HIV prevention programme is now operating at scale with 83 project sites, 169 drop-in centres and 619 STI clinics across 20 districts in Karnataka. Between January 2005 and March 2011, the number of female sex workers estimated to have regular contact with the programme (i.e. contacted at least once in a year) increased from 7,995 to 51,171, and the proportion of the total estimated FSW population reached each month increased from approximately 40% to over 85% (Figure 3). Over 900 peer educators are currently employed across Karnataka, distributing approximately three million free condoms per month. Since the HIV prevention programme began in 2004, more than 100,000 FSWs have been contacted at least once by the programmes, and more than 88,000 FSWs have accessed a project STI clinic. An average of 50,000 FSWs are contacted by peer educators each month, with > 17,000 FSWs visiting a project STI clinic each month (Figure 4).

The process of community mobilisation has resulted in a sharp increase in the number of FSWs becoming members of sex worker organisations (community-based organisations, self-help groups and community committees), from approximately 8,000 in January 2007 to 37,000 by October 2009 (Figure 4). In addition, by October 2009, approximately 2,500 FSWs were members of local HIV programme committees.

### Engagement with policy makers

By October 2009, District AIDS Committees had been established in each of the 18 districts where the programme was being implemented, and 178 government heads of departments had attended FSW and HIV sensitization and awareness workshops. District AIDS Committee membership of FSWs, including those living with HIV/AIDS, has been a key aspect of the empowerment process for these communities, and has provided them with a legitimate forum to raise their concerns and needs.

### Addressing stigma and discrimination

**Figure 5:**
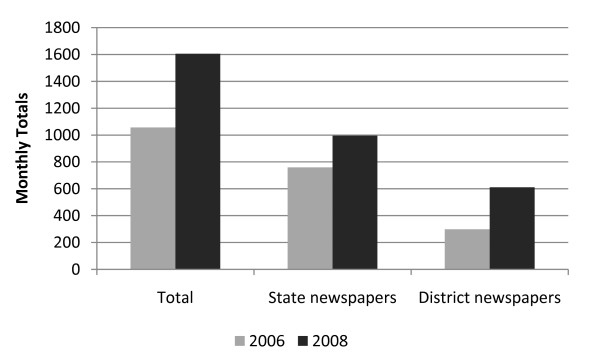
**Monitoring of news reports on HIV/AIDS and sex work, 2006-2008**.

Monitoring of news stories in 2006 and 2008 has shown a 50% increase in the number of media reports on HIV/AIDS and FSWs, from 1,057 in 2006 to 1,606 in 2008, with a doubling in the number of reports in district level newspapers, from 298 in 2006 to 611 in 2008 (Figure 5). News coverage of issues related to sex workers and people living with HIV/AIDS (PLWHAs), including FSW empowerment, increased from 375 reports in 2006 to 535 news reports in 2008, and news reports which included quotes or stories from FSW community representatives increased from 89 to 170. Meanwhile, the proportion of negative news reports focusing on "criminal" activities, violence, raids and arrests against FSWs fell from 11% in 2006 (117/1057) to 4% in 2008 (70/1606).

### Addressing violence and harassment

By August 2011, a total of 13,594 police personnel (12,786 males and 808 females), representing 61.5% of the police force, had received HIV/AIDS sensitisation and awareness training. In addition, in the 34 months between January 2007 and October 2009, a total of 4,600 rights violations against FSWs by the police and other sources (usually clients or other sex partners) were reported, with crisis management teams supporting sex workers to redress 92% of these, through meetings with the perpetrators or legal actions.

### Addressing social inequity

**Figure 6:**
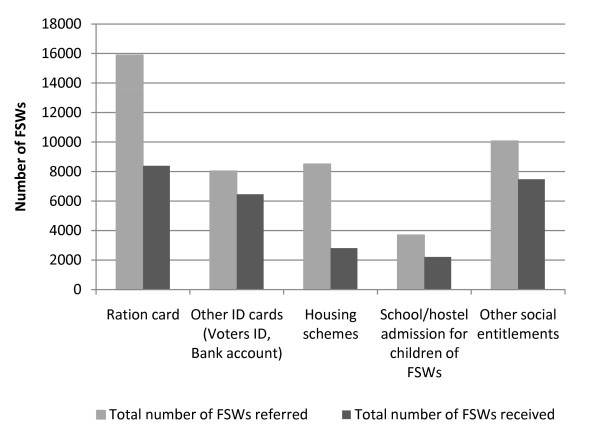
**Cumulative number of female sex workers assisted by the programme to access government social entitlements, 2006-2009**.

In the four years between 2006 and 2009, a total of 46,194 FSWs were referred for government social entitlements, with 27,355 (59%) successfully receiving these by the end of 2009. This included 15,891 FSWs referred for food ration cards, 8,029 for other ID cards (e.g. voter's identification, bank account), 8,509 for subsidized housing, and 3,694 children for subsidized school or hostel admission. By December 2009, 8,392 sex workers had received ration cards, 6,462 had received identification cards, 2,811 had been allocated subsidized housing, and 2,208 children had been provided support for enrolling in school (Figure 6).

## Discussion

HIV preventive interventions focused on vulnerable groups such as sex workers have traditionally targeted individual behaviour only, with impediments to success frequently including structural factors beyond the scope of these programmes [5,6]. In this paper, we have described a structural approach to HIV prevention which sought to change those context-specific social, economic, political and environmental factors determining HIV risk and vulnerability, as part of a large-scale HIV prevention programme in Karnataka state. We focused on addressing structural factors identified by the FSW community as contributing to their HIV vulnerability and, following collectivisation and community mobilisation of FSWs, worked in partnership with policy makers, secondary stakeholders (police, journalists and human rights lawyers), and primary stakeholders (FSWs themselves) to address issues of stigma and discrimination, violence and harassment, and social inequity facing the FSW community.

Previously we have reported significant increases in condom use, reductions in HIV and STI rates and reductions in violence against FSWs in Karnataka [16,21,25,26]. In this study we have reported that the HIV prevention programmes have supported FSWs to re-dress over 90% of reported incidents of violence and harassment against them. In addition, we have presented evidence of widespread FSW mobilisation and collectivisation, substantial increases in the numbers of FSWs gaining access to government social benefits, and substantial changes in the reporting of HIV/AIDS and sex work in district and state level newspapers. The impact of the various components of a structural intervention on reducing HIV vulnerability can be difficult to assess [1]. This is particularly true of structural barriers which are more distal to HIV risk, such as stigma and discrimination of FSWs by the general population, compared with more proximal factors such as sexual violence or micro-environmental factors affecting the conditions and resources of individuals (e.g. living conditions, food availability and education). Elsewhere, studies have demonstrated strong evidence of the detrimental impact on HIV risk of structural factors such as violence [32,36,43,44], poverty [45-47], and stigma and discrimination [48-51]. In addition, some studies have sought to address structural barriers contributing to HIV/STI risk [52,53], although there have been few studies demonstrating the direct impact of structural interventions on HIV risk among vulnerable populations [11,12,54]. Indeed, although the 100% condom programme in Thailand and the Sonagachi programme in Kolkata have both reported reduced or low rates of HIV among their target populations, without a randomised control trial design, it is difficult to directly assess the impact of the structural intervention components of these programmes on HIV prevalence [7,9,10,37,55].

Although we have presented evidence here suggesting that we are managing to successfully address some of the structural barriers identified as important by the FSW community, it is not possible for us to tease out from the data available, which components of our structural intervention have been most important in contributing to the reductions in HIV/STI rates. We are planning to conduct qualitative and process evaluation studies with FSWs to help in this respect. Evidence from studies elsewhere suggests that increased social capital at both the individual and community level could be important in reducing vulnerability to HIV [56-58]. In addition, evidence from elsewhere suggests that had we focused solely on individual behaviour change and not addressed the context-specific structural barriers contributing to the HIV vulnerability of FSWs, it is unlikely our HIV prevention programming would have been as successful [1,10,11].

There were several challenges to implementing this structural intervention, and lessons learned. One lesson is that it is important to develop the vulnerability reduction strategies from programme inception, as risk and vulnerability go hand in hand. In addition, vulnerabilities faced by FSWs are very contextual, and thus the involvement of sex workers in defining the structural drivers or factors causing vulnerability is crucial. For example, we found that while addressing violence was more important for certain FSW typologies and in certain geographic areas, for other FSWs improving social equity was more important. Addressing structural drivers is a long term intervention and requires patience from the donor and the implementer. Furthermore, any structural intervention work comes into direct conflict with power structures, which can sometimes lead to short-term increases in violence, discrimination and inequity. It is important that there is solidarity among the FSW population to be prepared for this, and that the supporting agencies provide maximum support to FSWs during this time. Finally, measuring the success or impact of structural interventions is complex, and the results of these interventions are not always consistent (for example, levels of reported violence can initially increase following a violence intervention due to violent reactions from established power structures, and increasing capability in violence reporting by FSWs) [25].

There are several limitations to a study such as this one. The absence of a precise baseline profile is a clear limitation, but it was not possible for us to conduct studies with the FSW community until trust had been established and the HIV prevention programme had begun. From our discussions with FSWs it is clear that prior to the programme commencing, no support systems (e.g. crisis management teams) or social entitlements were available to them, and condom availability was low [19]. The lack of cohort data makes it difficult for us to examine changes to individual behaviour over time, or to unpack the impact of the various structural interventions on changes to individual behaviours or STI/HIV rates. In addition, as most of the newspapers in Karnataka are not available online, the tracking of news reports is currently conducted manually, and may be subject to reporting error. Finally, it was beyond the scope of this paper to examine the cost and cost-effectiveness of this structural intervention, but such evaluations of the programme overall have been reported elsewhere, with results suggesting that this HIV prevention programme is not costly compared with other HIV prevention programmes, and that it is cost-effective [59,60].

## Conclusions

Taken together, we maintain that stigma and discrimination, violence and harassment, and social equity issues are all critical concerns of this FSW population, and all needed to be addressed, to enable vulnerable women to become sufficiently empowered to adopt the safer sexual behaviours which are required to respond effectively to the HIV epidemic. Although measuring the impact of a structural intervention is complex, addressing broader structural barriers contributing to HIV vulnerability as part of HIV prevention programming is not only possible, but is key to reducing the vulnerability of women.

## List of abbreviations

AIDS: acquired immune deficiency syndrome; CBOs: community-based organizations; DAC: district AIDS committee; FSWs: female sex workers; HIV: human immunodeficiency virus; KHPT: Karnataka health promotion trust; NGOs: non-governmental organizations; PLWHAs: people living with HIV/AIDS; STIs: sexually transmitted infections.

## Competing interests

The authors declare that they have no competing interests.

## Authors' contributions

VG, PB and HLM designed and oversaw local implementation of the intervention programme. TSB performed most of the analyses and wrote the first draft of the paper. CFAR performed the analyses on media coverage and contributed to writing the paper. SM, RW and JFB were involved in the study design, and contributed to writing the paper. SI and BMR designed and supervised the routine monitoring information systems. SM designed the study and contributed to writing the paper. All authors have read and approved the final manuscript.

## Pre-publication history

The pre-publication history for this paper can be accessed here:

http://www.biomedcentral.com/1471-2458/11/755/prepub
